# A Mobile App for Student Veterans With Co-Occurring Academic and Psychiatric Concerns: Pilot Usability and Feasibility Study

**DOI:** 10.2196/93871

**Published:** 2026-09-11

**Authors:** Erin Dawna Reilly, Lisa N Mueller, Donna M Crossman, Hannah L Grigorian, Shannon W Schrader, Megan M Kelly, Katarina Bernice, Marsha L Ellison

**Affiliations:** 1 Veterans Integrated Service Network 1 Mental Illness Research, Education, and Clinical Center Veterans Affairs Bedford Healthcare System United States Department of Veterans Affairs Bedford, MA United States; 2 Department of Psychiatry and Behavioral Sciences University of Massachusetts Chan Medical School Worcester, MA United States; 3 Psychology Department Veterans Affairs Bedford Healthcare System United States Department of Veterans Affairs Bedford, MA United States; 4 Jesse Brown Veterans Affairs Medical Center United States Department of Veterans Affairs Chicago, IL United States; 5 Veterans Integrated Service Network 1 Mental Illness Research, Education, and Clinical Center Veterans Affairs Connecticut Healthcare System United States Department of Veterans Affairs West Haven, CT United States; 6 Department of Psychiatry Yale University School of Medicine New Haven, CT United States

**Keywords:** higher education, mental health, mobile application, pilot trial, usability, veterans

## Abstract

**Background:**

Over 600,000 student veterans use military benefits to pursue higher education annually, with 75% enrolled as full-time students. Advancing their education is an important step toward improving their employment, financial, and social opportunities. However, student veterans also experience high rates of co-occurring psychiatric concerns like substance use disorder (SUD), posttraumatic stress disorder (PTSD), or depression. For these students, additional psychoeducation, support, and guidance may be required. To support their goals, a mobile app with targeted content for student veterans managing common psychiatric concerns could be particularly helpful.

**Objective:**

The primary aim of this pilot study was to iteratively revise and assess usability, acceptability, and feasibility of the VetEd mobile app and study procedures. The secondary aim was to explore preliminary changes from preapplication to postapplication use in academic functioning and community reintegration.

**Methods:**

This study used a mixed methods approach; pilot usability testing and iterative revisions were completed across 3 waves of participants (wave 1=4; wave 2=4; wave 3=5; total n=13). Usability was measured using the System Usability Scale (SUS), and acceptability was measured using the Acceptability E-Scale (AES). App use feasibility was collected through app engagement data from the 4 weeks of mobile app use. Study feasibility was assessed by rates of recruitment, retention, and survey completion. Secondary, exploratory measures to assess potential VetEd use outcomes included academic self-efficacy, perceived ability to cope with educational barriers, and community reintegration. Participants were also given the opportunity to complete an exit interview to provide additional feedback.

**Results:**

Study feasibility was high, with greater than projected rates of recruitment (4-5 per month), retention (13/13, 100%), and survey completion at all 3 time points (100%, 85%, and 100%, respectively). Overall, 85% (11/13) of both usability and acceptability scores were above the predefined benchmarks. Completion of assigned VetEd activities was high, with participants completing, on average, 90.8% of requested app activities. Secondary exploratory outcomes in academic self-efficacy, coping with educational barriers, and community reintegration were nonsignificant but showed feasibility for use in future fully powered randomized VetEd trials. Exit interviews highlighted that VetEd was easy to use, included pertinent information for both academic and mental health functioning, and had vital information on connecting to additional resources.

**Conclusions:**

Pilot findings suggested that the VetEd mobile app is usable and acceptable as an asynchronous resource for student veterans managing psychiatric concerns. Exit interviews suggested that veterans found app content useful, with relevant information and resources to support their academic functioning. Additional research is needed to assess its effectiveness, implementation, and impact on student veterans nationwide.

**Trial Registration:**

ClinicalTrials.gov NCT05344092: https://clinicaltrials.gov/study/NCT05344092

## Introduction

Higher education can be key to future vocational success, as people with higher levels of education are more likely to be employed [1], earn more money over their lifetime [2], and exhibit increased resilience to unemployment [3]. Education level is also positively associated with psychological and physical outcomes, including increased happiness [4] and better physical health status [5]. For US veterans, the opportunity to obtain these positive outcomes from higher education is fundamental, with financial support for college often endorsed as a primary motivator for enlisting in the military [6]. Since the Post-9/11 GI Bill was first introduced in 2009, more than 1.4 million veterans, service members, and their families have used these benefits to fund their educational pursuits [7]. On an economic level, educational attainment for veterans generally increases their career prospects and subsequent earnings [7]. Additionally, educational success is associated with greater quality of life, as veterans with at least some college education exhibit better employment opportunities, stable housing, and family and social roles [8,9].

While veterans bring multiple strengths to academic settings, transitioning to the culture of higher education can be complicated by challenges that impact daily functioning and academic performance. One such domain is psychological functioning, as veterans experience mental health difficulties at elevated rates and have higher rates of suicide compared with civilians [10,11]. These veterans can also face difficulties related to addictive (eg, alcohol), cognitive (eg, attention), or emotional/behavioral concerns (eg, posttraumatic stress disorder [PTSD] or depression), leading to lower enrollment rates [12] and slower degree attainment [13]. Student veterans with mental health conditions can also have less positive educational outcomes due to challenges adjusting to campus life [14] and interpersonal difficulties with faculty [15]. These challenges can impede veterans’ successful adjustment to civilian life and downstream vocational success.

Regrettably, many veterans with mental health concerns neither report problems nor seek treatment [11]. Even for veterans seeking support, other barriers exist, including time, transportation, and lack of resource knowledge [16,17]. Given these challenges, the US Department of Veterans Affairs (VA) has invested in many staff-delivered programs for academic support and mental health treatment, using a catalog of supported education (SEd) options through compensated work therapy/supported education (CWT/SEd), Veteran Integration to Academic Leadership (VITAL), and VA mental health clinics [18]. While preliminary evidence from SEd pilots such as VITAL-SEd [17] has shown promise in assisting veterans with mental health concerns to achieve educational success [19,20], barriers to these staff-delivered programs still exist (eg, transportation, resources, and availability).

Although the VA has mobile apps for veteran support, training, and resources, there is no mobile app specific to student veterans’ needs and resources. Digital health interventions are especially relevant to student veterans given their comfort with technology [21] combined with high rates of time-intensive commitments beyond coursework (eg, caregiving and nonacademic work) [22] that can reduce the use of in-person programming. Additionally, anonymous, digital options for psychoeducation and support may be of particular interest to veterans with mental health concerns, who often do not access interventions due to potential stigma or perceived negative consequences [21,23,24]. Consequently, student veterans could benefit from an asynchronous mobile app providing a centralized virtual location for support, resources, and psychoeducation.

The VetEd mobile app was developed to increase educational information, academic skill acquisition, and access to both health and academic support for veterans and was evaluated in a pilot usability and feasibility trial. The objectives of the pilot were to (1) evaluate study methodology feasibility and iteratively revise the VetEd app across 3 waves (n=4-5 per wave; total n=12-15) by assessing app usability, use, content acceptability, and satisfaction data, and (2) explore changes in secondary outcomes from before app use to after app use for student veterans on academic self-efficacy, ability to address educational barriers, and community reintegration.

## Methods

### Ethical Considerations

This study was approved by the institutional review board of the VA Bedford Healthcare System, Bedford, Massachusetts (#1679573-10), and the requirement for written informed consent was waived. Informed consent was obtained during the virtual baseline appointment; study staff and the potential participant reviewed the study information sheet verbally, and after this review, participants verbally provided consent to participate in study activities. Data were deidentified. This manuscript follows the CONSORT (Consolidated Standards of Reporting Trials) reporting guidelines for pilot and feasibility trials (Multimedia Appendix 1).

### Study Trial Design and Recruitment

This study conducted a single-arm iterative evaluation and refinement pilot of the mobile app VetEd, while also collecting outcome data from before app use to after app use (4 weeks). All potential participants were prescreened by phone to determine whether basic study eligibility criteria were met. If still eligible, they were scheduled for a baseline session to complete the informed consent process, confirm eligibility, and complete all baseline surveys (approximately 2 hours total). Participants were then provided with the app and login information for VetEd via encrypted email, with instructions on how to use the app at home over the 4-week study period. Additional feedback was provided through an optional exit interview (approximately 30 minutes) following app use.

### Participant Recruitment

Participants were recruited over 3 months via mailers, community outreach events, and flyers/brochures posted across the VA Bedford Healthcare System campus to assess mobile app use during their fall semester. Inclusion criteria included the following: (1) currently a veteran enrolled in at least 2 courses in a higher education setting, (2) current mental health disorder (ie, *DSM-5* [*Diagnostic and Statistical Manual of Mental Disorders* {Fifth Edition} psychiatric diagnosis confirmed via VA medical record review and with participant self-reporting currently struggling with the mental health disorder), (3) competent to provide informed consent, (4) owns a smartphone capable of supporting mobile apps, (5) willing and able to read in English, (6) aged 18 years or older, and (7) self-report struggling with course activities. Exclusion criteria included: (1) not currently enrolled as a student, (2) current or recent (within 1 month of study entry) moderate or severe *DSM-5* alcohol or substance use disorder (SUD) as assessed via the Structured Clinical Interview for *DSM-5* Disorders, Research Version (SCID-5-RV [25]), (3) cognitive impairment that would interfere with participation, and (4) severe suicidal ideation or a psychiatric hospitalization within the past month. Veterans were not required to be in active treatment for any mental or physical health disorders. However, they were required to be enrolled in VA care for medical record reviews for inclusion and exclusion criteria. Additionally, veterans with moderate to severe SUD within the past month were excluded from trial participation and study activities in order to prioritize their SUD care within VA. Those meeting this level of SUD severity on the SCID-5-RV would instead have consults placed for VA SUD services.

### Intervention

#### VetEd Mobile App

**Figure 1:**
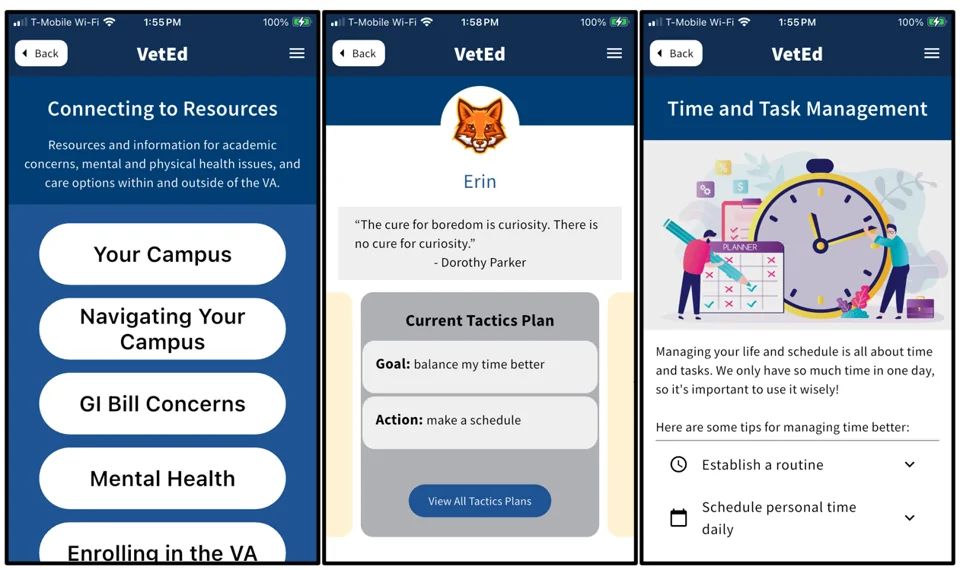
Images from the VetEd mobile app.

**Figure 2:**
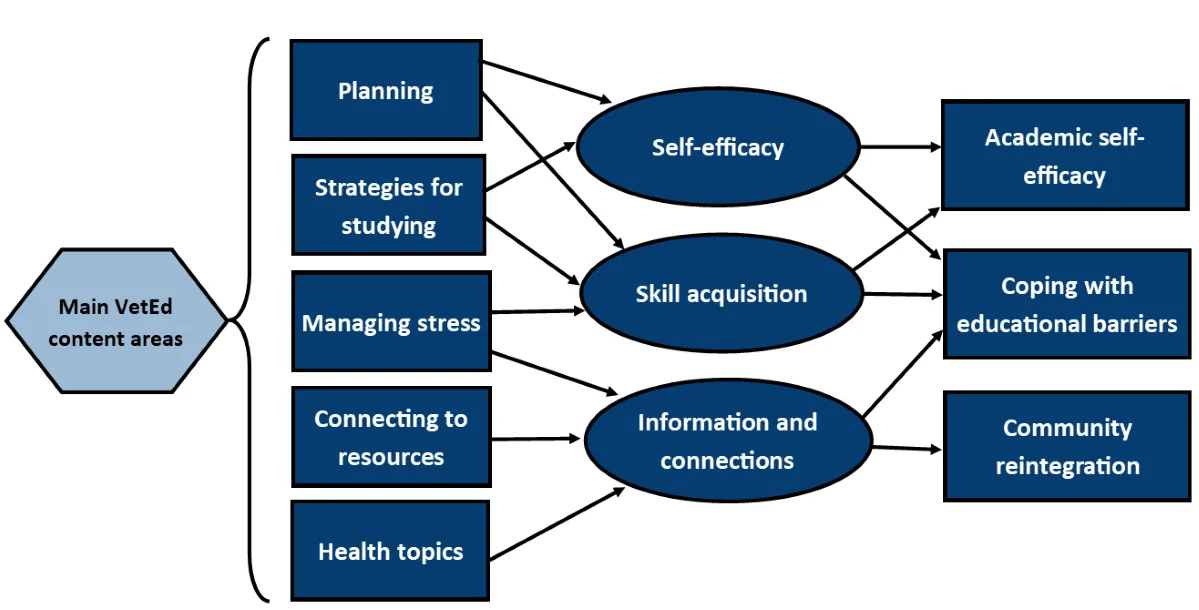
Theoretical model for the VetEd mobile app and outcome targets.

The purpose of VetEd is to support academic success, tailored to veterans struggling with additional mental health concerns such as substance use, mood disorders, and PTSD. The initial VetEd mobile app content (Figure 1) was developed primarily from existing manualized veteran SEd treatments (ie, VITAL-SEd [19,26]), with additional content added from online VA student resources [27] and primary intervention components across 5 content areas of student development (Table 1). The framework and content were derived largely from the SEd model, which assists individuals with psychiatric disabilities or mental health challenges in accessing, participating in, and completing postsecondary or secondary education. Similar to SEd, VetEd is rooted in the principles of psychosocial rehabilitation and the model of personal recovery [28,29]. Aligning with this person-centered, strengths-based recovery model, VetEd specifically presents 5 content areas to target the overarching SEd purpose aim of choosing, getting, and keeping to their educational path by (1) promoting self-efficacy so veterans can independently define their academic and campus needs and set goals for their achievement, (2) helping students acquire skills by learning and applying new academic and coping strategies, and (3) providing information and connections to resources based on the veteran’s perceived needs (eg, information on mental health counseling, existing college resources like obtaining academic accommodations, and Post-9/11 GI Bill coverage). VetEd’s objective is to strengthen these 3 domains of academic and health management. These improvements are theorized to foster academic success by building educational self-efficacy, assisting with community reintegration, and improving students’ ability to navigate academic barriers (secondary outcomes; Figure 2).

Following the creation of the initial app content and wireframes, a group of subject matter experts and 4 small prepilot focus groups were conducted with student veterans (number per focus group=3, 2, 4, and 3) to review the preliminary app and gather feedback on student veteran needs, interests, and perspectives for additional improvements. Using this combined information, the final testable VetEd was created in collaboration with Veterans Health Administration (VHA) and Geisel Software, Inc, for this pilot.

**Table 1:** Main VetEd mobile app areas and content.

Main content areas	Description	Available content
Planning	Activities to help increase self-efficacy and orient to the student role by reflecting on educational and career goals and organizing academic choices into a roadmap with actionable education goals. Users were able to track and report on their progress and goal completion in this section.	Education tactics planning Calendar
Strategies for studying	Short, informational activities and modules focused on the skills necessary to effectively complete coursework and class activities.	Time and task management Effective study tips Improving your memory Knowing when to get additional help
Managing stress	Activities and guidance for mental health issues, stress management, and coping strategies for difficult thoughts and feelings. This included passive content presentation as well as guided video exercises (eg, mindfulness meditations).	School stress management Dealing with stressful thoughts Planning for pleasant activities
Connecting to resources	Resources and information for academic concerns, mental health issues, and Veterans Benefits Administration/GI Bill benefits information modeled off the VA^a^ Inquiry Routing & Information System. This included in-app resource lists, hyperlinks to VA resources, and phone numbers for additional VA consultations.	Your campus Navigating your campus GI bill concerns Mental health services Enrolling in the VA
Health topics	Education about managing common mental and physical health issues that can impact academic goals/impacts, including brief psychoeducation and hyperlinks to resources for care/management.	TBI^b^ and cognitive issues Substance use issues PTSD^c^ Depression Anxiety Chronic pain Insomnia

^a^VA: US Department of Veterans Affairs.

^b^TBI: traumatic brain injury.

^c^PTSD: posttraumatic stress disorder.

#### Pilot Testing Procedures

During the 4-week study, participants were asked to complete 2 in-app tasks per week. Instructions were provided specifically about which to access in weeks 1, 2, 3, and 4 via encrypted email from a study staff member. There was no other human involvement during the intervention phase. Participants were explicitly told they could also access app content beyond these activities; however, to obtain feedback on a range of content and interactive elements, we requested completion of 8 specific activities from all participants (Table 2). Natural use of the mobile app and information on use of other app areas were not collected by the app in this initial pilot. Participants completed surveys before, during, and after the pilot to evaluate the following areas: (1) app usefulness: whether users can successfully complete designated tasks on the app, (2) effectiveness: the ability to accomplish assigned educational app tasks quickly and easily, (3) learnability: the ability to meet predetermined app navigation goals (eg, accessing and inputting information into the education roadmap), (4) satisfaction: users’ feelings and opinions about the app related to their educational functioning and alignment with mental health needs, and (5) engagement: initial and sustained interaction between the user and mobile app. After each wave of testing, the app version was frozen for 2 weeks, and the investigative team worked with the programming team to improve the app. Participants were compensated US $80 for the baseline assessment, US $20 at the midpoint survey at week 2, and US $90 for the final survey (week 4). Participants were compensated immediately after completing each assessment time point, and all compensation was provided via Amazon gift cards. Participants were not compensated based on mobile app use.

**Table 2:** Requested weekly mobile app activities.

Week	Requested activity	Estimated time (minutes)
1	Create an education tactics plan action item Read over “time and task management tips”	4 3
2	Create a pleasant activities list Review the “enrolling in the VA^a^” page	4 2
3	Create a calendar item Read over “dealing with stressful thoughts”	4 3
4	Read over 1 health topic of interest under “health topics” Record at least 1 item in the “your campus resources” section	3 3

^a^VA: US Department of Veterans Affairs.

### Outcomes

#### Demographics

Demographic measures included veterans’ self-reported information on current age, sex, race, ethnicity (eg, Hispanic or Latino), service era, service branch, service-connected disability and rating, employment information, financial condition, living situation, and current relationship status. A medical record review collected information on diagnosed mental and physical health disorders. Finally, to describe baseline mental health functioning of the sample, we also administered the 9-item Patient Health Questionnaire-9 (PHQ-9 [30]) to report on depressive symptoms and the 20-item PTSD Checklist for *DSM-5* (PCL-5 [31]) for PTSD symptom severity. This information was used to describe the demographics and health-related characteristics of the student veteran sample.

#### Primary Outcomes: Feasibility, Usability, and Acceptability

The primary outcomes for this pilot trial were usability, acceptability, and feasibility of the mobile app and feasibility of the study protocol. Mobile app use data were directly collected from the app, including the percentage of usability testing tasks completed (Table 2). The System Usability Scale (SUS [32]), a 10-item measure scored on a 5-point Likert scale to assess human-computer interaction, was the primary usability outcome. A SUS score above 68 (range 0 to 100) was set as the cutoff for adequate usability per wave, as this is regarded as above average. The SUS generates a subjective evaluation score using a globally accepted scale to understand if the system in its current form is sufficiently usable. Additionally, the Acceptability E-Scale (AES [33]), a 6-item validated measure scored on a 5-point scale, was used to indicate acceptability and satisfaction with app content overall, with a cutoff of 80% (total score 24, range 6-30). Both the SUS and AES were administered at the midpoint (week 2) and postuse assessment (week 4), with week 4 scores used as the primary outcome end point.

#### Secondary Exploratory Outcomes: Educational and Reintegration

As exploratory outcomes, preliminary information related to academic self-efficacy, perceived confidence in coping with educational barriers, and community reintegration was collected at prestudy and poststudy evaluations. In line with educational research suggesting that factors beyond grade point average (GPA) can be stronger predictors of educational success and graduation [34], we specifically assessed these areas using the following scales.

The Academic Self-Efficacy Scale (ASES [35]) is an 8-item rapid assessment instrument to assess respondents’ self-efficacy related to areas aligned with academic achievement, such as time management, taking notes, taking tests, and general academic ability. Each statement is scored on a scale from very untrue (score 1) to very true (score 7). Scores range from 8 to 56, with higher scores indicating greater reported academic self-efficacy. In this sample, internal consistency was above the acceptable range (Cronbach α=0.94).

The Coping With Education Barriers Subscale (CEBS [36]) includes 21 items to assess participants’ confidence in overcoming internal and external educational barriers. Each statement is rated by the participants’ degree of confidence that they could overcome the potential educational barrier, from not at all confident (score 1) to highly confident (score 5). Higher scores (range 21-105) reflect greater perceived confidence in overcoming common educational barriers, and internal consistency for this sample was high (Cronbach α=0.95). In addition, 2 single-item questions were used to assess general perceptions of barriers related to education, revised from the 2-item subscale [37]. Rated on a scale from strongly disagree (score 1) to strongly agree (score 5), the two items were (1) “In general, I think that there are many barriers facing me as I try to achieve my educational goals,” coded as “educational barrier severity,” and (2) “In general, I think that I will be able to overcome any barriers that stand in the way of achieving my educational goals,” coded as “educational barrier self-efficacy.”

The Military to Civilian Questionnaire (M2C-Q [38]) is a 16-item measure that assesses dimensions of community reintegration related to productivity (in education and work), social relations, community engagement, and perceived meaning in life, self-care, and leisure. Items are rated on a scale from no difficulty (score 0) to extreme difficulty (score 4), with higher scores indicating greater difficulties with community reintegration and ranging from 0 to 64. Internal consistency for this sample was high (Cronbach α=0.91).

### Statistical Methods

To address our primary outcomes, we planned to evaluate and describe study feasibility (rate of recruitment, retention, attrition, survey completion, and VetEd app task completion), as well as VetEd usability (SUS) and acceptability (AES; program feedback and suggestions). Each usability testing wave included 4 to 5 participants, based on prior research suggesting that approximately 80% of usability problems can be detected using a sample of 4 to 5 participants per wave [39]. Our study hypothesized that the AES and SUS ratings would increase across waves of app revisions and that, at a minimum, by the third wave, they would reach the benchmark scores of 68 on the SUS and 24 on the AES. Feasibility and engagement data were analyzed by measuring the proportion of individuals who successfully completed the 4-week app testing, with at least 5 completed activities (of the requested 8, 2 per week), or 63% of the program completed, in line with the mean number of completed virtual intervention activities found in previous mobile intervention clinical trials for chronic mental and physical health conditions [40]. Additional usability, acceptability, and usefulness information was also provided by the qualitative exit interviews offered to every participant following their month of app use. Participant feedback was transcribed and coded using a modified consensual qualitative research approach [41,42] (coders EDR and HLG). An inductive approach, specifically, a conventional content analysis [19], was used, in which coders allowed domains and codes to emerge from the text after immersing themselves in the data. Discrepancies between coders were resolved via coder dialogue and consensus-building meetings, with the use of an independent auditor (ie, an external staff member not otherwise associated with the project) as needed. The consensus reached during these discussions was used to refine the guiding codebook (eg, domains and themes) and complete cross-case analysis. The modified consensual qualitative research process specifically included the following 6 phases of thematic analysis: reviewing a sample of the qualitative data for potential preliminary domains (n=2 interviews), consensus meetings to discuss and generate initial code domains, reviewing the prior interviews (n=2) to search for initial themes within domains, revising domains and themes as needed during consensus meetings, reviewing for themes across all remaining interviews (n=4), defining and naming themes within domains, including having domains and themes audited by an independent staff member, and producing the final results using the constructed domains and themes from common trends across all interviews. For final results, response themes mapped to each domain were reviewed for frequency. Given the small sample size and to avoid overinterpretation of themes, the reported and interpreted themes reflect factors within each domain that met criteria for typical frequency (eg, >50%, or at least 4/6 of the sample) according to modified consensual qualitative research procedures [41].

We assessed academic functioning and community reintegration measures before app use and after app use for each student veteran specifically to (1) characterize the sample, (2) assess for possible changes, and (3) test the feasibility of these measures in anticipation of a larger, future randomized controlled trial. This assessment included the exploratory aim that app use may lead to improvements in our hypothesized underlying mechanisms for improved educational success—ASES, perceived ability to complete course tasks (CEBS), or improvements in community reintegration (M2C-Q). To investigate this, we conducted nonparametric, within-subject statistical analyses between before app use and after app use scores on these measures using Wilcoxon signed-rank tests, as is appropriate for small-sample testing and thus outcomes that may not be normally distributed. Due to the goal and design of this pilot, we were not powered to detect significant changes on secondary outcomes, and analyses were intended as descriptive and exploratory.

## Results

### Participant Flow and Sample Description

**Figure 3:**
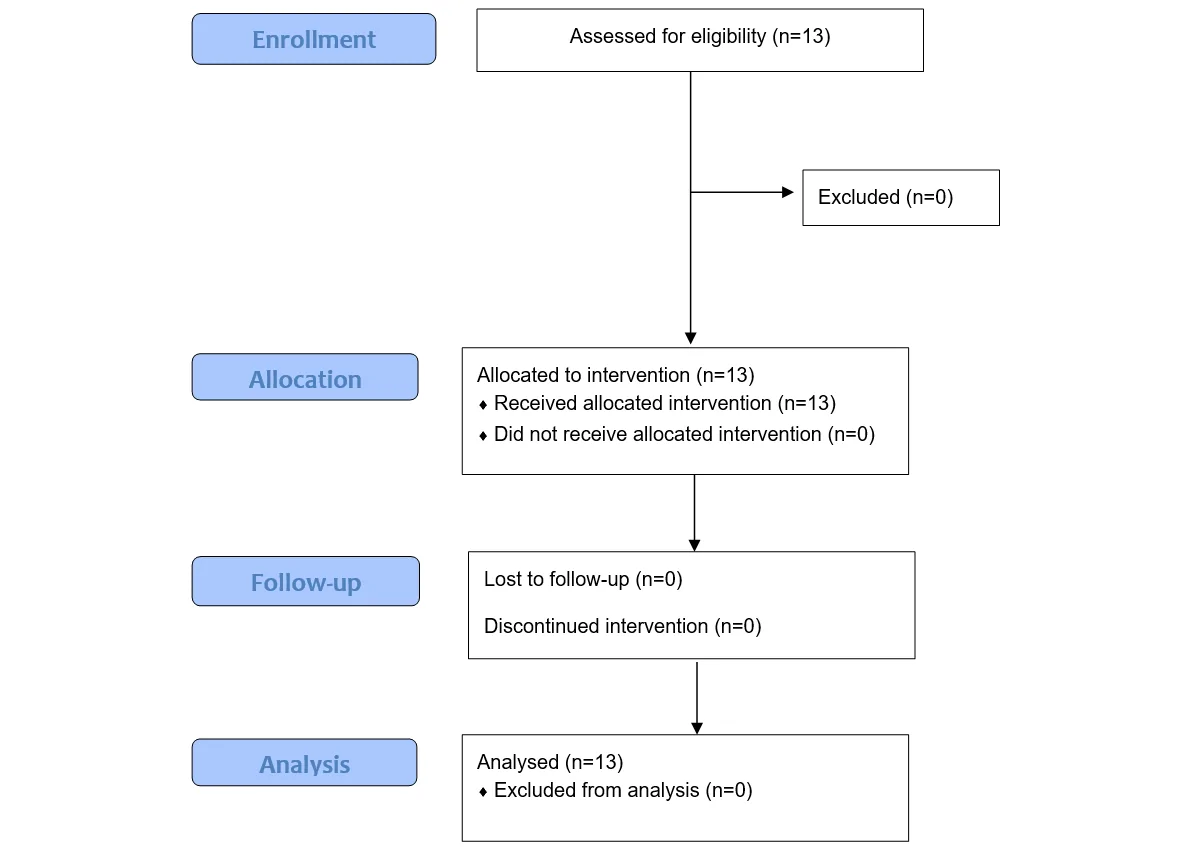
CONSORT (Consolidated Standards of Reporting Trials) flow diagram for the pilot study.

The mean age of the pilot usability sample (n=13) was 32.4 (SD 7.8) years. Most of the sample was male (9/13, 69.2%), White (10/13, 76.9%), and not Hispanic or Latino (10/13, 76.9%). All participants (13/13, 100%) reported having served after September 2001 and reported having a VA service-connected disability rating. In terms of employment, only 3 participants were currently working for pay in addition to attending school (3/13, 23.1%). Additionally, a little more than half (7/13, 53.8%) reported having at least some difficulty making ends meet financially. Finally, most participants were living with a family member or romantic partner, including a parent/in-law (6/13, 46.2%). Figure 3 provides participant flow, and Table 3 provides a full demographics summary.

**Table 3:** Pilot usability testing participant demographics (N=13).

Characteristic			Value
Age (years), mean (SD)			32.4 (7.8)
**Gender, n (%)**			
	Men	9 (69.2)	
	Women	4 (30.8)	
**Race, n (%)**			
	American Indian or Alaskan Native	1 (7.7)	
	Black or African American	1 (7.7)	
	White	10 (76.9)	
	Multiracial	1 (7.7)	
**Ethnicity, n (%)**			
	Hispanic or Latino	3 (23.1)	
	Not Hispanic or Latino	10 (76.9)	
**Service era^a^** **, n (%)**			
	September 2001 or later	13 (100)	
	August 1990-August 2001	1 (7.7)	
**Service branch^a^** **, n (%)**			
	Army	2 (15.4)	
	Air Force	1 (7.7)	
	Navy	5 (38.5)	
	Marines	5 (38.5)	
	National Guard	1 (7.7)	
**Service-connected disability rating (%), n (%)**			
	50- 60	1 (7.7)	
	70-90	5 (38.5)	
	100	7 (53.8)	
**Employment^a^** **, n (%)**			
	Working for pay	3 (23.1)	
	Working as a volunteer	1 (7.7)	
	Not working and not actively looking for work	1 (7.7)	
	Not working but actively looking for work	1 (7.7)	
	Unable to work	3 (23.1)	
	Retired	2 (15.4)	
	Homemaker or unpaid caretaker	1 (7.7)	
	Declined to answer	1 (7.7)	
**Financial condition, n (%)**			
	In over my head	2 (15.4)	
	Tough to make ends meet but keeping my head above water	3 (23.1)	
	Occasionally have some difficulty making ends meet	3 (23.1)	
	Able to make ends meet without much difficulty	4 (30.8)	
	Very comfortable and secure	1 (7.7)	
**Living situation, n (%)**			
	Living with parent(s) or in-laws	6 (46.2)	
	Living with spouse or partner	2 (15.4)	
	Living with friends	1 (7.7)	
	Living with their (or their partner’s) children	1 (7.7)	
	Living with other relatives/family	1 (7.7)	
	No one else, living alone	3 (23.1)	
**Current relationship status, n (%)**			
	Single, never married	6 (46.4)	
	Divorced	3 (23.1)	
	Married	2 (15.4)	
	In a relationship, not married	2 (15.4)	

^a^Participants could choose multiple categories.

In terms of baseline academic and mental health functioning, 69.2% (9/13) reported agreement with the statement “In general, I think that there are many barriers facing me as I try to achieve my educational goals.” In terms of co-occurring mental health conditions, all participants had at least 2 current, diagnosed mental health disorders, with the most common being major depressive disorder (11/13, 84.62%), PTSD (7/13, 53.85%), generalized anxiety disorder (6/13, 46.15%), and attention-deficit/hyperactivity disorder (5/13, 38.46%); a smaller proportion also reported managing chronic pain (3/13, 23.08%). Average PHQ-9 (12.3, SD 8.1) and PCL-5 (41.1, SD 24.8) scores were high, with 11/13 (84.62%) above the minimum depression cut point (>5) and 69.2% (9/13) above the PCL-5 score of 33, indicating probable PTSD.

### Primary Outcomes: Feasibility, Usability, and Acceptability

#### Feasibility

Measures of study feasibility, app usability, and intervention acceptability were assessed from baseline (week 0) to the primary final assessment (week 4) time point. The rate of monthly recruitment was higher than expected (anticipated 2 to 3 recruits per month), with approximately 4 to 5 veterans per month. Pilot testing was conducted over 5 months, with 2 weeks between each wave of usability testing to complete VetEd revisions requested by participants. The total rate of study retention following consent was 100% (13/13). Survey assessment completion was high across all time points, with completion rates of 100% (13/13) at the baseline (week 0) survey, 85% (11/13) at the midpoint (week 2) survey, and 100% (13/13) at the postintervention (week 4) survey.

Engagement with VetEd for study feasibility was assessed by evaluating the proportion of individuals who successfully completed the 4-week app testing protocol (ie, at least 5 completed activities of the requested 8 activities, or 63% of the program). In wave 1, users completed an average of 75% of activities, with 2 participants completing 8/8 activities and 2 participants completing 4/8 (50%). In wave 2, all participants met the cutoff, with 3 participants completing all activities and 1 participant completing 80% (6/8). In wave 3, all 5 participants completed 100% of the requested weekly in-app activities. In total, most users (11/13) met or exceeded the predefined completion threshold of 5 or more in-app activities completed that would suggest the in-app requested study activities were feasible for users to complete during their participation and to provide informed feedback. All participant feedback was used and interpreted, regardless of meeting the target individual “use” rate of 5/8 completed activities, to ensure breadth of feedback, especially from users with potentially more negative or neutral app experiences.

#### Usability and Acceptability

In Table 4, we report scores on the usability testing outcome measures for each iterative testing wave. Between each wave, 2 weeks were allocated to make user-suggested revisions. After wave 1, major changes to VetEd were made following user suggestions related to (1) the creation and inclusion of an in-app “site map,” (2) redoing portions of the calendar to allow for different appointment recording options, and (3) addressing usability concerns related to the profile page. Between waves 2 and 3, the only usability suggestions were related to programming fixes to address link issues (eg, fixing broken links to VA resources). The main quantitative usability measure was the SUS, and usability scores were initially higher than anticipated, with our data reaching the original benchmark of 68 by wave 1 (74.4, SD 5.2) and holding steady across wave 2 (78.1, SD 7.2) and wave 3 (76.0, SD 13.6) following small revisions to the app. The mobile app also met the acceptability AES benchmark (ie, a total score of 24) on the first wave of testing (24.3, SD 5.2), which also held across wave 2 (28, SD 1.8) and wave 3 (24.2, SD 4.2). Overall, 85% of SUS scores were above the predefined benchmark of 68 (11/13); only 2/13 scores (15%) were below the SUS benchmark. Similarly, 85% of AES scores (11/13) were at or above the cutoff score of 24, with 2/13 (15%) below the cutoff score.

**Table 4:** Mean usability and acceptability scores.

Measure	Wave 1 (n=4), mean (SD)	Wave 2 (n=4), mean (SD)	Wave 3 (n=5), mean (SD)	Total (N=13), mean (SD)
System Usability Scale	74.4 (5.2)	78.1 (7.2)	76.0 (13.6)	76.2 (9.2)
Acceptability E-Scale	24.3 (5.2)	28 (1.8)	24.2 (4.2)	25.4 (3.7)

Exit interview feedback was collected to further evaluate acceptability and usability. A total of 46% (6/13) of participants completed these interviews at week 5, with 2 participants completing interviews per iterative usability testing wave. Feedback was coded across 5 general domains: motivations to use VetEd, use and usability of the app, VetEd content and programming feedback, future dissemination to veterans, and perceived app impact on educational functioning and health management (reported in the Secondary Exploratory Outcomes: Mental Health, Academic Functioning, and Community Reintegration section). Exit interview participants reported multiple themes related to motivation to use the app, with the most common being to support their academic success. Within the domain of motivations to use VetEd, specific themes included hoping the app would help with (1) educational task improvement (eg, to aid in memory issues), (2) mental health management (eg, PTSD and TBI), and (3) managing school-adjustment issues (eg, lack of social support and feeling disconnected from other students). As one student veteran explained:

I realized that a lot of things I feel veterans struggle with is benefits or [that] these programs normally exist to help with something. They just don't know. And so a lot of times I feel like people might think there's only one solution like, oh, I have to do ‘this’. But normally there's various solutions, and again it's just something they don't know. So I found that would be something I would keep this app for and definitely use.

Participant 106, aged 30-35 years

Within this domain, veterans also typically noted the belief that the app could attenuate the negative impact of mental health issues on academics related to (1) poor attention, (2) stress, and (3) being disorganized. This aligned with use patterns, as interviewees most commonly reported personally benefiting from the app in the areas of (1) course organization, (2) learning “new skills” like study tips, or (3) exploring additional academic and mental health information.

So the biggest piece of information that I never knew, I believe one of the points with the strategies for studying, it's like after class take 15 minutes to recall everything that’s happened. You know, at least then you don't need to cram textbooks 24/7 to shove information that you might not need. That was the point where I was like, WOW, did it make sense! And I practice it. Test it from start to right now, this semester, and it has been better for me. Because the past two semesters I was struggling immensely. Not just with my PTSD, but like getting adjusted to a new school. You know, I'm in a unknown area. I'm not from XXXX, you know. I don't have family friends here. But I do have one thing, and that's education, and that's what I focused on and seeing that being simplified helped a lot.

Participant 105, aged 25-30 years

Participants also explored barriers and facilitators to app use within the domain of use and usability of the app. Commonly reported barrier themes included technical concerns (eg, login issues), difficulty finding content, and the belief that the app as a VA-branded product could be a “negative thing.” On the other hand, one common facilitator theme included the perception that the app reminding veterans of the “VA” would be a positive facilitator and increase confidence in the resource. Other commonly reported facilitator themes of VetEd use included (1) its design encouraging use (eg, “simple,” “well-organized,” “straightforward,” “easy to navigate,” and “user friendly”) as well as (2) the ease of accessing content related to resources and information (eg, study strategies and tips and information on mental health issues), with one user highlighting the convenience of in-app content linkages:

But the user experience was good. There's this whole thing where, like, you shouldn't have to click three times if you can just click one time, right? […] I mean, we're all doing stuff on our phone now, so it's having it available and through the app, it’s more sensible. Oh, it's got resources there, it's all about tying to the resource, which it does.

Participant 101, aged 40-45 years

In terms of the VetEd content and programming feedback domain, all participants reported positive themes related to the graphic design and layout, with the most typical themes being that the app was (1) simple and easy to use (eg, “not distracting”) and (2) had a very good graphic design (eg, with “a nice color scheme”). In terms of privacy and security, typical themes that arose included (1) having no concerns about privacy or security, (2) feeling that a login was unnecessary, and (3) wanting to continue to keep data “local” (eg, only stored in the phone app). Themes in this domain also mapped directly onto content feedback and future suggestions; all interviewees reported enjoying content across the 5 main app areas, describing it as “good content,” “well rounded,” and “comprehensive.” Specific themes that emerged in relation to content suggestions were (1) adding a search feature, (2) tailoring recommendations based on a user’s demographics or geographic location, and (3) inclusion of a forum or way to connect student veterans to one another for help and support, with one person stating:

Another thing too is, I don't know if it's possible, but if there was a way to like help link them to like other veteran communities. […] So I think being able to help connect you to these other places where you can learn more or find other people that are in similar situations would help you know with, like, especially that loneliness.

Participant 106, aged 30-35 years

Finally, feedback within the future dissemination to veterans domain provided suggestions on advertising and timing of the app more broadly. All interviewed participants reported that they would recommend the app to other veterans and be likely to use it in the future if it was publicly available. Specific dissemination themes included (1) explaining the app’s purpose in advertising materials, (2) tailoring and advertising specific content to the app user based on demographics (eg, going to school in a certain state), and (3) promoting the app early in a veteran’s transition to civilian life, with one veteran stating:

It should be a requirement that the VA has to mention that they should probably get it. Yeah, I think the sooner the better. I think a lot of people could be benefiting from this app. Like I said, if I had it four years ago, I think that my time in school would have been a lot easier.

Participant 113, aged 30-35 years

### Secondary Exploratory Outcomes: Mental Health, Academic Functioning, and Community Reintegration

This trial was not sufficiently powered to detect significant changes in secondary outcomes after 4 weeks of app use. Instead, academic self-efficacy, ability to address educational barriers, and community reintegration were all evaluated for study measure feasibility and described from preintervention to postintervention to explore potential changes and describe trends (Table 5). ASES total scores showed no significant difference (*z* score=–0.84; *P*=.40; Cohen *d*=–0.11, 95% CI –0.66 to 0.44) from preintervention (median 40.0, IQR 31.5-45.5) to postintervention (median 45.0, IQR 25.5-50.0). Descriptively, 69% (9/13) of student veterans’ scores showed greater perceived academic self-efficacy after using VetEd. On the CEBS, there were no significant changes in total scores (*z* score=–0.11; *P*=.92; Cohen *d*=–0.07, 95% CI –0.62 to 0.47) from preintervention (median 77.0, IQR 62-92) to postintervention (median 83.0, IQR 54-96); descriptively, 62% (8/13) of participants reported greater perceived confidence in addressing common educational barriers after using the VetEd mobile app on the CEBS. In terms of changes in general perception of self-efficacy in addressing educational barriers, educational barriers self-efficacy single-item scores showed increases but no significant difference (*z* score=–1.63; *P*=.10; Cohen *d*=–0.48, 95% CI –1.05 to 0.106) between preintervention (median 3.0, IQR 3.0-5.0) and postintervention (median 5.0, IQR 3.0-5.5). Finally, on the M2C-Q, there were no significant changes (*z* score=–0.42; *P*=.68; Cohen *d*=0.21, 95% CI –0.35 to 0.75) from preintervention (median 3.21, IQR 2.5-3.9) to postintervention (median 3.13, IQR 1.7-4.3); descriptively, 46% (6/13) of student veterans reported fewer perceived difficulties with community reintegration on the M2C-Q following use of VetEd.

Exit interviews provided additional functional outcome information within the domain of perceived app impact on educational functioning and health management. Typical positive impact themes were (1) the usefulness of having a “central location” to connect to academic, mental health, and benefit resources within and outside the VA; (2) helping them stay organized and plan for courses; (3) providing strategies for educational improvements (eg, study tips, taking notes, and time management); and (4) providing psychoeducation and treatment options for mental health issues (eg, PTSD and SUD). Three themes also emerged as reasons why the app might not be beneficial for a veteran personally, including (1) portions of the app were less useful due to overlap with other educational support services they were receiving (eg, VITAL); (2) some of the app seemed generic, with information that was not relevant to them (though all noted it could be helpful to other veterans); and (3) parts of the app (ie, “navigating your campus”) were unhelpful for students later in their academic experience. No participant reported adverse study events or that the app had a negative impact on their academics, mental health, or overall well-being, with one veteran noting:

I think this app would be beneficial to the people that are just getting out of the military doing the like VA transition and going to school. Because, I've been in school for the past four or five years. I'm about to graduate in December. And it helped me. I think it would have helped me a lot more four years ago if I had it right away when I got out and I was going to school. But I think it's going to be awesome moving forward for the newer veterans that are coming out and going to school.

Participant 110, aged 30-35 years

**Table 5:** Exploratory secondary outcome scores.

Measure	Wave 1 (n=4), median (IQR)		Wave 2 (n=4), median (IQR)		Wave 3 (n=5), median (IQR)		Total (N=13), median (IQR)	
	Preintervention	Postintervention	Preintervention	Postintervention	Preintervention	Postintervention	Preintervention	Postintervention
ASES^a^	37.5 (29.8-40.8)	32.5 (22.5-47.3)	31.5 (18.5-49.8)	38.0 (21.3-52.5)	41.0 (39.0-52.5)	47.0 (40.0-51.0)	40.0 (31.5-45.5)	45.0 (28.5.-50.0)
CEBS^b^	70.5 (61.0-79.3)	78.0 (54.5-92.5)	80.5 (48.3-100.8)	74.0 (45.5-101.8)	85.0 (61.5-94.0)	83.0 (60.5-98.0)	77.0 (62.0-92.0)	83.0 (54.0-96.0)
EdB-SE^c^	3.0 (3.0-4.5)	4.0 (3.0-5.0)	4.5 (2.3-6.0)	5.5 (2.7-6.0)	5.0 (2.5-5.0)	5.0 (3.5-5.5)	3.0 (3.0-5.0)	5.0 (3.0-5.5)
M2C-Q^d^	2.7 (1.3-3.5)	1.7 (1.2-3.8)	3.4 (1.8-4.3)	3.7 (1.5-4.3)	3.6 (3.1-4.0)	3.6 (2.7-4.0)	3.2 (2.5-3.9)	3.1 (1.7-4.3)

^a^ASES: Academic Self-Efficacy Scale.

^b^CEBS: Coping With Education Barriers Subscale.

^c^EdB-SE: Educational Barriers Self-Efficacy.

^d^M2C-Q: Military to Civilian Questionnaire.

## Discussion

### Primary Outcomes: Feasibility, Usability, and Acceptability Findings

Pilot findings supported all preregistered feasibility, acceptability, and usability outcomes. First, study procedures were highly feasible in terms of participant recruitment (nearly double the projected rate) and study assessment completion rates (100% final survey completion and study retention). Veterans reported that the VetEd app was highly usable and completed, on average, 90% of the requested mobile app tasks with high after-use app acceptability scores. This aligns with prior research on the use of VA mobile apps by veterans with mental health concerns, which has reported high levels of app interest, though varying rates of engagement [43]. These strong pilot study findings underscore veteran interest and potential engagement with both study activities and the mobile app, suggesting that further dissemination and evaluation testing of VetEd is warranted. This was also reflected in qualitative feedback, which suggested a positive response across all major domains (ie, motivation to use, usability, perceived usefulness, and specific VetEd app content), with student veterans noting the content, layout, programming, and graphic design were intuitive, simple, and “easy to understand.” Future app revisions suggested by users included adding a search function, notifications, and more content tailored to the user based on their needs and demographics. Future VetEd iterations, as well as other VA apps to assist veterans with mental and physical health issues, should consider the growing importance of user-specific tailored content for increased acceptability and usefulness.

### Exploratory Outcomes: Mental Health, Academic Functioning, and Community Reintegration

The primary purpose of this pilot usability study was to assess the feasibility, usability, and acceptability of the study protocol and mobile app, and our sample size was determined for this primary aim. Secondary aims were used to assess measurement feasibility and describe differences in changes from preusability testing to postusability testing on outcome measures for academic functioning, educational self-efficacy, and community reintegration. Feasibility of these potential future outcome measures was high, with completion rates of all 3 surveys (ASES, CEBS, and M2C-Q) above 80% at each time point. In terms of secondary outcomes as potential signal-detection data, none showed significant changes between preintervention and postintervention. Descriptively, most student veterans (9/13, 69%) reported greater perceived academic self-efficacy, which is consistent with research suggesting that self-efficacy in higher education courses can be increased by providing support for skill and information acquisition both in person [44] or via mobile apps [45]. In addition, over half of the veteran app users reported increased confidence in their ability to cope with perceived educational barriers after using VetEd, suggesting there is a signal for the main purposes of the app (eg, to increase self-efficacy, academic skill sets, and get connected to resources for managing the health concerns that can be major barriers to success). Additionally, more than half of veterans reported increased perceived difficulty in community reintegration over the course of the 4-week pilot (ie, from the start to midpoint of their semester). This suggests that social and community reintegration may be a particular issue for these veterans and an important area for additional evaluation in student veteran work. Prior research has also highlighted common feelings of social isolation, lack of support, and loneliness among student veterans on college campuses [46]. Social connection may thus be a particularly important area for future trial measurement and app revisions (eg, resources on connecting veterans to peer support and programming [47,48]).

### Limitations and Future Directions

This study has additional limitations and strengths. First, as the purpose of this project was to conduct a single-arm open pilot trial of the mobile app, the study had limited statistical power; thus, secondary outcome findings related to academic functioning and quality of life should be interpreted with caution. This includes the limitation of a small number of participants per wave, such that changes in usability scores could have been due to usability improvements or an artifact of differences between participants (eg, high scores reflecting a wave with more technologically savvy users). A fully powered randomized controlled clinical trial should be conducted in the future to assess the usability and efficacy of VetEd on academic functioning, quality of life, and mental health outcomes. Second, all users engaged with the app at the very beginning of the fall semester for a finite time (4 weeks total), which limits information related to continued engagement and potential impact across the full semester. Additionally, users were explicitly instructed on what app activities needed to be completed per week and, although participants were not compensated for completion of app activities, they were compensated for study participation through survey completion. Both factors may have influenced VetEd use rates reported in this study, and consequently, in-app activity completion is not necessarily indicative of real-world use information. Future research should allow veterans to use the VetEd app for a full semester or longer to explore how users would naturally interact with a publicly available and unguided VetEd app.

The small and homogenous sample size of this pilot also further limits the generalizability of study findings. All participants were enrolled in the same geographic area and were connected to care at a VA hospital with multiple options for supported education and academic assistance that are unfortunately not available to all student veterans nationally (eg, VITAL). Relatedly, participants were all connected to VA with service-connected disabilities and compensation, which may have influenced both study engagement and perceived usefulness of the app given its emphasis on VA resources. Future research should not only expand the reach of VetEd beyond this area but also investigate the potential usefulness of the app for student veterans who are not VA–service-connected or VA-engaged and do not have these VA resources available, such as those who live in rural areas, are not service-connected, or are not engaged in VA services.

Finally, although a full review of future implementation of VetEd into the VA is beyond the scope of this pilot report, it is important to consider how future research can explore VetEd scalability, implementation, and integration into VA systems. The VetEd app meets the requirements to be posted in the VA Mobile App Store catalog [49], with all data collected locally, a login required for use, Health Insurance Portability and Accountability Act (HIPAA) compliance, no collection of protected health information or personally identifiable information, and compliance with Section 508 accessibility requirements. However, VetEd is currently hosted external to VHA (eg, the app is hosted by Geisel Software, Inc, via a contract with VHA) and has not been nationally disseminated. Future important areas for consideration prior to implementation will include (1) considerations for disseminating VetEd beyond VITAL- and service-connected student veterans, (2) a thorough review of maintenance costs and burden for VA hosting of VetEd, (3) establishing strategies for updating VetEd, and (4) collaboration with VA operational partners on integrating and connecting VetEd with current VA apps (eg, VA Health and Benefits app).

### Conclusions

Overall, this pilot project resulted in a tailored, iteratively refined mobile app for student veterans, providing a new option in the VHA mobile app portfolio to support academic success and higher education attainment for many veterans. Findings showed high usability and satisfaction with the VetEd app, while also providing promising initial qualitative results as to its potential usefulness. Future research is needed to assess the impact of VetEd on academic self-efficacy, coping with educational barriers, and community reintegration. With further additions and modifications, the VetEd app could be particularly useful for soon-to-be-discharged service members planning to pursue higher education, as well as student veterans. These results suggest the potential for VetEd to provide an accessible, acceptable, and useful option for veterans and student service members in community care to improve their educational outcomes.

## Appendix

Multimedia Appendix 1 CONSORT checklist.

## Abbreviations

AES: Acceptability E-Scale
ASES: Academic Self-Efficacy Scale
CEBS: Coping With Education Barriers Subscale
CONSORT: Consolidated Standards of Reporting Trials
CWT: compensated work therapy
DSM-5: Diagnostic and Statistical Manual of Mental Disorders (Fifth Edition)
GPA: grade point average
HIPAA: Health Insurance Portability and Accountability Act
M2C-Q: Military to Civilian Questionnaire
PCL-5: PTSD Checklist for DSM-5
PHQ-9: Patient Health Questionnaire-9
PTSD: posttraumatic stress disorder
SCID-5-RV: Structured Clinical Interview for DSM-5 Disorders, Research Version
SEd: supported education
SUD: substance use disorder
SUS: System Usability Scale
VA: US Department of Veterans Affairs
VHA: Veterans Health Administration
VITAL: Veteran Integration to Academic Leadership

## Data Availability

The datasets generated and analyzed during this study are not publicly available due to the security requirements of the US Department of Veterans Affairs but are available from the corresponding author on reasonable request. The authors will consider reasonable requests on a case-by-case basis, subject to compliance with the US Department of Veterans Affairs data-sharing agreements.
